# Supplementary material for: Metabolic Integration of Spectral and Chemical Cues Mediating Plant Responses to Competitors and Herbivores
Source: Plants (Basel). 2022 Oct 19;11(20):2768. doi: 10.3390/plants11202768 (PMC9609625; doi:10.3390/plants11202768)
Supplement: Supplementary file 1 [file plants-11-02768-s001.zip › plants-1939409-supplementary.pptx]

## Slide 1
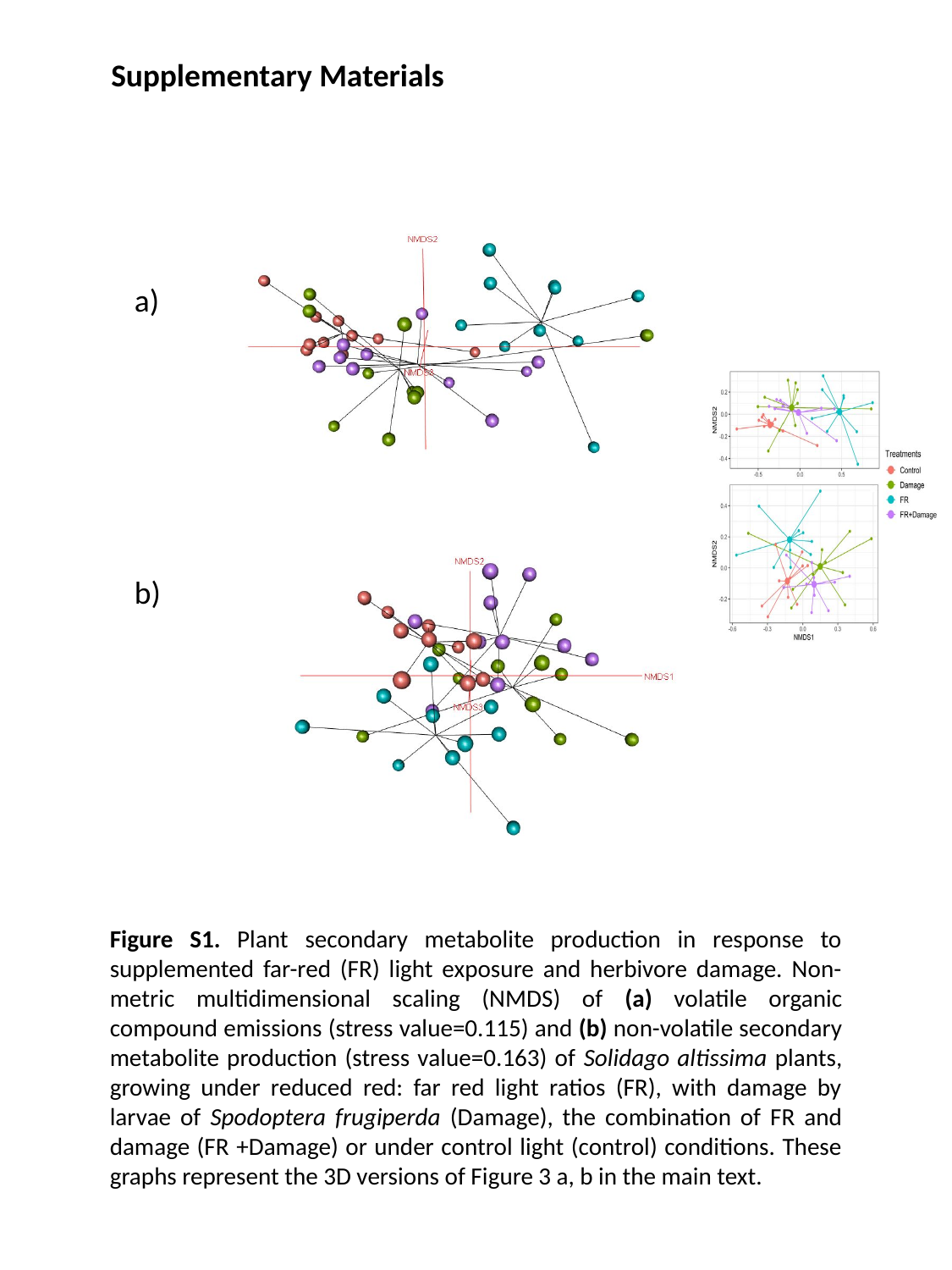

Supplementary Materials
a)
b)
Figure S1. Plant secondary metabolite production in response to supplemented far-red (FR) light exposure and herbivore damage. Non-metric multidimensional scaling (NMDS) of (a) volatile organic compound emissions (stress value=0.115) and (b) non-volatile secondary metabolite production (stress value=0.163) of Solidago altissima plants, growing under reduced red: far red light ratios (FR), with damage by larvae of Spodoptera frugiperda (Damage), the combination of FR and damage (FR +Damage) or under control light (control) conditions. These graphs represent the 3D versions of Figure 3 a, b in the main text.

## Slide 2
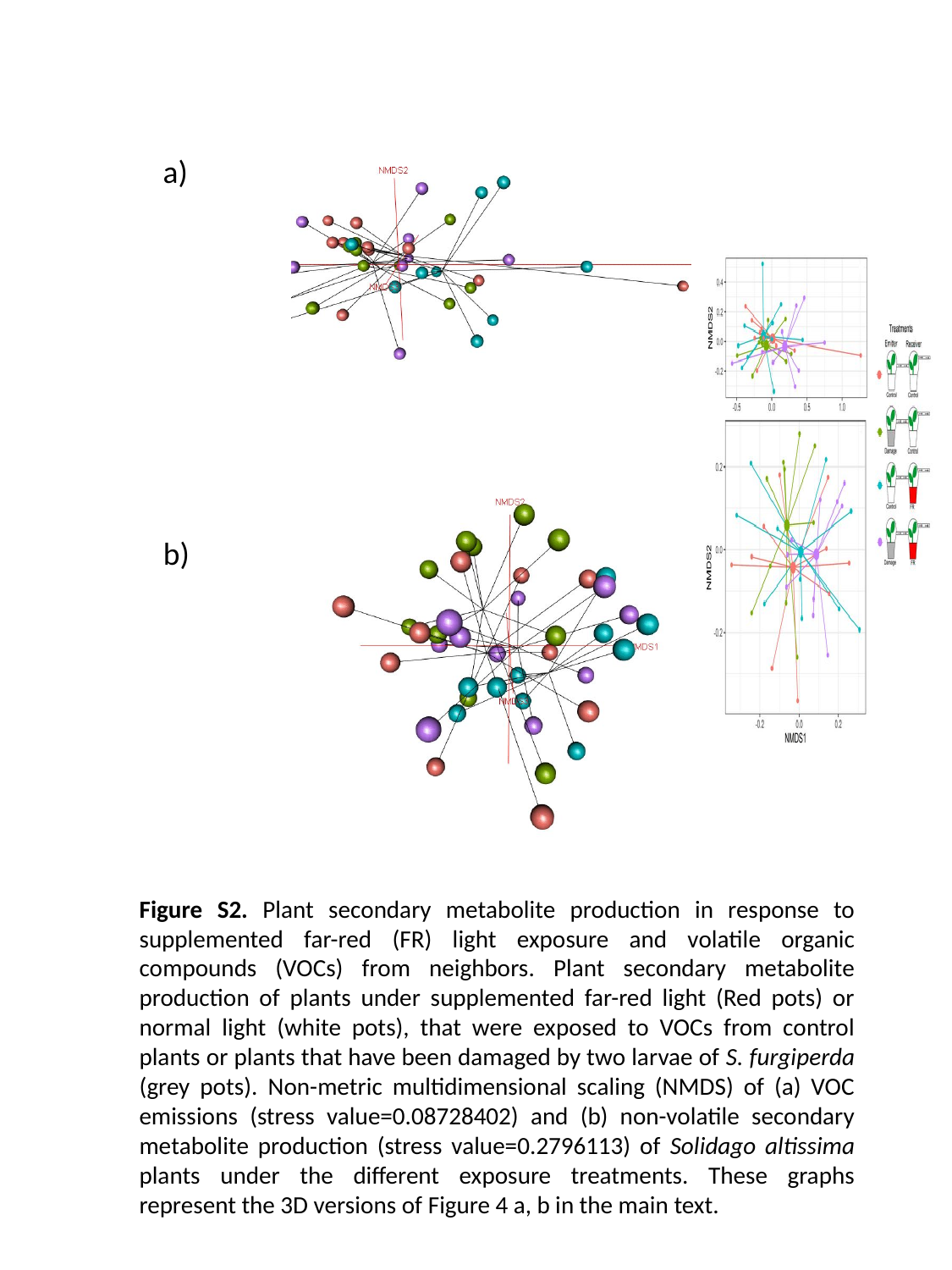

a)
b)
Figure S2. Plant secondary metabolite production in response to supplemented far-red (FR) light exposure and volatile organic compounds (VOCs) from neighbors. Plant secondary metabolite production of plants under supplemented far-red light (Red pots) or normal light (white pots), that were exposed to VOCs from control plants or plants that have been damaged by two larvae of S. furgiperda (grey pots). Non-metric multidimensional scaling (NMDS) of (a) VOC emissions (stress value=0.08728402) and (b) non-volatile secondary metabolite production (stress value=0.2796113) of Solidago altissima plants under the different exposure treatments. These graphs represent the 3D versions of Figure 4 a, b in the main text.
